# Interventional therapy of acute coronary syndromes in very old patient population and results of 2 years follow-up

**DOI:** 10.1186/s43044-023-00340-x

**Published:** 2023-02-22

**Authors:** Bedrettin Boyraz, Tezcan Peker, Alkame Akgümüş, Ahmet Balun

**Affiliations:** 1 Cardiology Department, Medicalpark Hospital, Mudanya University, Kırcaali, Fevzi Çakmak Cd. No:76, 16220 Osmangazi, Bursa Turkey; 2 Cardiology Department, Medical Faculty, Bandırma Onyedi Eylül University, Balıkesir, Turkey

**Keywords:** Acute coronary syndrome, Very old patient population, Two years follow-up, Interventional theraphy

## Abstract

**Background:**

Research on cardiovascular treatment options and prognosis in very old age groups of patients is warranted. In our study, we evaluated and followed up on clinical conditions on admission and comorbidities of patients older than 80 years who were admitted to our hospital with acute myocardial infarction and shared our findings.

**Results:**

A total of 144 patients were included in the study, with a mean age of 84.56 ± 5.01 years. No complications resulting in death or requiring surgery were observed in the patients. All-cause mortality was found to be related to heart failure, chronic pulmonary disease shock, and C-reactive protein levels. Cardiovascular mortality was correlated to heart failure, shock on admission, and C-reactive protein levels. No significant difference in mortality was observed between Non-ST elevated myocardial infarction and ST-elevation myocardial infarction.

**Conclusions:**

Percutaneous coronary intervention is a safe treatment option with low complication and mortality rates in very old patients with acute coronary syndromes.

## Background

Decades of research have focused on the prognosis of patients admitted to the hospital with acute coronary syndrome (ACS), especially non-ST elevated myocardial infarction (NSTEMI) and ST-elevation myocardial infarction (STEMI) [1–3]. The main objectives of these studies were to define the therapies that patients would most benefit from, identify which patients should be followed up in the intensive care unit, and inform patients and their relatives about the possible outcomes.

Many studies tend to exclude elderly patients. Furthermore, it is anticipated that elderly patients would benefit more in terms of the principle; patients with higher risk–benefit most. The term very old people is mostly used to describe people older than 80 years. Coexisting comorbidities increase with growing age. It is not uncommon for this age group to have multiple vascular comorbidities, such as cerebrovascular events, peripheral arterial disease, and coronary artery disease (CAD) [4–6]. The multivessel disease is a common finding on coronary angiography (CAG) of these patients [7, 8]. The intervention of culprit lesions is typically carried out in these patients; however, non-culprit lesions are medically treated compared to younger patients. The decision to perform surgery is made depending on the Synergy between PCI with Taxus and Cardiac Surgery (SYNTAX) score and the eligibility of lesions for percutaneous coronary intervention (PCI) [4, 9–11]. PCI is mostly the initial choice when the comorbidities and clinical conditions of patients are taken into account. This patient group is generally excluded from studies for the reasons like the delay in the diagnosis and treatment, administration of various drug therapies of the clinician’s choice, and patient-related issues such as incompatibility with drugs, presence of comorbidities, lack of nursing and exercise, and cessation of therapy because of operation. Because of all the aforementioned circumstances, the choice of therapy, benefits acquired by patients, and research evaluating long-term prognosis are lacking.

Long-term follow-up studies of elderly patients presenting with ACS are limited, these patients were not include in studies or are followed up for a short time (6 months) generally. According to current guideline recommendations, old age is not a contraindication for CAG. Conditions such as high troponin levels and deviation of ST-segment on electrocardiogram (ECG) are high-risk factors for patients and guidelines recommend early CAG to these patients. However, this situation is often overlooked in this group of patients due to age and additional comorbidities, and patients are followed up medically. In our study, we aimed to present both short-term and long-term follow-up results of aggressive interventional treatment in very elderly patients presenting with acute myocardial infarction (MI).

## Methods

This is a single-center and retrospective study. Patients older than 80 years who had been admitted to our hospital with a diagnosis of NSTEMI or STEMI between the years 2017 and 2019 were included. All study procedures involving human participants were in accordance with the ethical standards of the institutional and national research committee and with the 1975 Helsinki declaration and its later amendments or comparable ethical standards. A total of 184 patients were included. Guideline-directed diagnosis and therapy for acute myocardial infarction were performed in accordance with the most recent guidelines of the European Society of Cardiology. Patients who had been diagnosed with acute MI were grouped into STEMI and NSTEMI according to the guidelines [12]. All the patients underwent CAG. A total of 20 patients with troponin elevations, which are secondary to other causes, were excluded. Another group of 5 patients with recent surgical intervention or PCI in the previous 90 days and 5 patients with active malignancy were also excluded. Comorbidities and medications were noted on admission. Blood samples were analyzed and recorded. Calculation of the GRACE score depends on age, systolic blood pressure, heart rate, creatine, presence of cardiac arrest, ST-segment deviation, elevated cardiac biomarkers, and Killip classification on admission. Patients with no sign of heart failure are Killip class 1; those with jugular venous distention and rales on basal pulmonary segments are Killip class 2; those with pulmonary edema are Killip class 3; and those with cardiogenic shock are Killip class 4 [13–15]. The SYNTAX scores of the patients after CAG were calculated and recorded [9–11]. Stenoses between 70 and 95% were classified as severe and that > 95% as critical. The follow-up procedures were conducted in our hospital. Routine health care monitoring after acute myocardial infarction was executed in accordance with the current guidelines. Data from a small patient group that was not followed up in our facility were collected via telephone. The mortality records of deceased patients, including time and reason of death, were analyzed and saved. Target lesion revascularization (TLR), target vessel revascularization (TVR), and non-TVR were recorded. Ten patients who could not be reached for follow-up procedures were excluded. Continuous variables were implied as means ± standard deviations. Frequencies were noted as numbers and percentages. Cox proportional regression analysis was performed for uni- and multivariate regression analyses and noted as odds ratio (OR) and 95% confidence interval (CI). Following univariate analysis, the patients with *p* < 0.1 were switched to multivariate analysis, and forward regression was conducted. *p* < 0.05 was accepted for statistical significance. The Kaplan–Meier survival analysis was used for patients with STEMI and NSTEMI. SPSS (v.22.0; IBM Corp., Armonk, NY, USA) was used for the analysis of the data gathered.

## Results

A total of 144 patients were included in the study, with a mean age of 84.56 ± 5.01 (minimum 80 and maximum 106) years. Of the participants, 68 (47.2%) were male and 76 (52.8%) were female. One hundred fourty (97.2%) of the patients had hypertension. Fifty-four (37.5%) patients had STEMI and 90 (62.5%) had NSTEMI on admission. All the patients underwent CAG, and PCI was performed on 116 (80.6%) of these patients. The median GRACE score of the patients was 147.54 ± 20.93, and the mean ejection fraction (EF) on echocardiography was 47.43 ± 9.99. The laboratory findings, the baseline characteristics and scores of the patients are presented in Table 1.

**Table 1:** Baseline characteristics and laboratory data

Parameters	Values
Age	84.56 ± 5.01
Gender (Male %)	68 (47.2%)
Atrial fibrillation (%)	12 (8.3%)
Diabetes mellitus (%)	44 (30.6%)
History of coronary artery surgery (%)	9 (6.2%)
Dislipidemia (%)	36 (25%)
Hypertension (%)	140 (97.2%)
Coronary artery disease (%)	24 (16.7%)
Heart failure with reduced ejection fraction (%)	21 (14.6%)
Chronic pulmonary disease (%)	57 (39.6%)
Chronic renal failure (%)	17 (11.8%)
Previous stroke (%)	12 (8.3%)
ST-segment elevation myocardial infarction	54 (37.5%)
Percutaneous coronary intervention	116 (80.6%)
Shock on admission	10 (7%)
GRACE score	147.54 ± 20.93
Ejection fraction	47.43 ± 9.99
Hemoglobin (g/dl)	13.14 ± 1.86
Creatine (mg/dl)	1.14 ± 0.55
White blood cells (10 × 9/l)	9.55 ± 3.52
C-reactive protein (mg/dl)	31.43 ± 38.35

Regarding complications, one patient had major bleeding from the gastrointestinal system that required transfusion after PCI, with total recovery. No major access site complications requiring surgical intervention, such as pseudoaneurysm and giant hematoma, were observed. One patient had coronary perforation during the procedure, but it was managed with sequential balloon inflations and medical intervention, and the patient recovered. Four (2.8%) of the patients encountered contrast-induced nephropathy, and without dialysis, their creatine levels returned to the normal range with medical therapy alone.

Mortality was observed in 37 (25.7%) of the patients during follow-up, and 15 (10.4%) of these were cardiac deaths. Six (4.2%) had TLR, 6 had TVR, and six had non-TVR. Angiographic and follow-up data are provided in Table 2.

**Table 2:** Angiographic and follow-up data

Parameters	Values
SYNTAX score	16.83 ± 9.93
Vessels more than 70% occlusion	0: 16 (11.1%) 1: 70 (48.6%)
	2: 44 (30.6%) ≥ 3: 14 (9.7%)
Vessels more than 95% occlusion	0: 39 (27.1%) 1: 82 (52.9%)
	2: 19 (13.2%) ≥ 3: 4(2.8%)
Chronic total occlusion	13 (9%)
Median follow-up time (Months)	24.50 ± 11.61
Cardiac cause deaths	15 (10.4%)
All cause deaths	37 (25.7%)
In-hospital deaths	10 (6.9%)
6th months deaths	24 (16.7%)
First year deaths	29 (20.1%)
Two years deaths	34 (29.6%)
Target lesion revascularization	6 (4.2%)
Target vessel revascularization	6 (4.2%)
Non-target vessel revascularization	6 (4.2%)

Ten (6.9%) patients had in-hospital mortality. No significant difference in in-hospital mortality was observed between NSTEMI and STEMI (5 vs. 5; *p* = 0.39). The patients with in-hospital mortality had higher C-reactive protein (CRP) levels (75.75 vs. 14.85; *p* < 0.001) and lower EFs (35 vs. 50; *p* < 0.001) compared to those without. Multivariate analysis revealed heart failure with reduced EF (HFrEF), shock on admission, and CRP levels to be predictors of in-hospital mortality. The results are presented in Table 3.

**Table 3:** In-hospital mortality univariate and multivariate regression analysis

	Univariate			Multivariate		
	Odds ratio	95% CI	*p*	Odds ratio	95% CI	*p*
Grace score	1.04	1.02–1.07	0.000			
Chronic renal failure	4.98	1.40–17.64	0.013			
Heart failure	8.78	2.47–31.13	0.001	6.96	1.93–25.03	0.003
Atrial fibrillation	4.71	1.21–18.23	0.025			
Ejection fraction	0.93	0.90–0.97	0.001			
Shock on admission	18.53	5.23–65.67	0.000	17.97	4.39–73.59	0.000
Cardiac arrest on admission	7.88	0.99–62.26	0.050			
Urea	1.01	1.00–1.02	0.000			
Creatine	2.63	1.49–4.64	0.001			
Alanine amino transferase	1.005	1.003–1.007	0.000			
Aspartate amino transferase	1.004	1.002–1.006	0.000			
White blood cells	1.17	1.09–1.25	0.000			
C-reactive protein	1.01	1.009–1.02	0.000	1.02	1.01–1.03	0.000

Regression analyses were executed regarding all-cause and cardiovascular deaths. All-cause mortality was found to be related to HFrEF, chronic pulmonary disease, shock, and CRP levels. Cardiovascular mortality was found to be correlated to HFrEF, shock, and CRP levels. Data are summarized in Tables 4 and 5.

**Table 4:** Cardiac cause mortality univariate and multivariate regression analysis

	Univariate			Multivariate		
	Odds ratio	95% CI	*p*	Odds ratio	95% CI	*p*
Age	1.04	0.95–1.14	0.32			
GRACE score	1.03	1.01–1.05	0.000			
Diabetes mellitus	2.64	0.95–7.29	0.06			
Chronic renal failure	2.82	0.90–8.89	0.07			
Heart failure	5.55	2.00–15.38	0.001	4.48	1.59–12.56	0.004
Atrial fibrillation	4.19	1.33–13.18	0.01			
Chronic pulmonary disease	3.15	1.07–9.22	0.03			
Ejection fraction	0.94	0.91–0.97	0.000			
Shock on admission	12.45	3.88–39.99	0.000	10.30	3.05–34.69	0.000
Cardiac arrest on admission	5.52	0.72–42.06	0.09			
Urea	1.01	1.007–1.02	0.000			
Creatine	2.05	1.20–3.50	0.008			
Alanine amino transferase	1.004	1.002–1.007	0.000			
Aspartate amino transferase	1.003	1.001–1.006	0.003			
White blood cells	1.16	1.09–1.24	0.000			
C-reactive protein	1.01	1.007–1.02	0.000	1.01	1.007–1.02	0.000
Number of vessels with severe stenosis(> 70%)	1.19	0.67–2.12	0.54			
Number of vessels with critical stenosis (> 95%)	1.40	0.77–2.56	0.26			
Gender	0.59	0.21–1.67	0.32

**Table 5:** All-cause mortality univariate and multivariate regression analysis

	Univariate			Multivariate		
	Odds ratio	95% CI	*p*	Odds ratio	95% CI	*p*
Age	1.05	1.00–1.12	0.05			
GRACE score	1.02	1.01–1.04	0.000			
Chronic renal failure	2.71	1.28–5.75	0.009			
Heart failure	5.48	2.83–10.63	0.000	3.98	1.98–7.98	0.000
Atrial fibrillation	2.64	1.10–6.34	0.03			
Chronic pulmonary disease	3.61	1.81–7.19	0.000	2.77	1.36–5.67	0.005
Ejection fraction	0.95	0.93–0.97	0.000			
Shock on admission	7.08	2.44–20.50	0.000	8.53	2.73–26.69	0.000
Urea	1.01	1.008–1.02	0.000			
Creatine	1.80	1.23–2.64	0.002			
Alanine amino transferase	1.004	1.002–1.006	0.001			
Aspartate amino transferase	1.003	1.002–1.005	0.000			
Hemoglobin	0.86	0.73–1.02	0.09			
WBC	1.15	1.08–1.22	0.000			
C-reactive protein	1.01	1.009–1.02	0.000	1.01	1.01–1.02	0.000
Troponin	1.00	1.00–1.00	0.08			
Number of vessels with severe stenosis (> 70%)	1.15	0.80–1.66	0.44			
Number of vessels with critical stenosis (> 95%)	1.08	0.71–1.63	0.71			
Gender	0.93	0.49–1.78	0.83			

No significant difference in mortality was observed between NSTEMI and STEMI (NSTEMI—OR 1.46; 95% CI 28.58–34.32 and STEMI—OR 1.73; 95% CI 24.76–33.11; *p* = 0.38).

## Discussion

Mortality and morbidity rates of very old patients group have been found to be higher in recent studies, which corroborate our findings. However, the complication rates in our study were not high. According to the results of numerous studies, medical treatment is the preferred strategy in this age group considering complications. Our study demonstrated lower complication rates than predicted. Mortality rates were high when evaluated according to the GRACE score.

Our study demonstrated similar mortality and morbidity rates for patients with NSTEMI and STEMI, which is correlative to previous studies. Gender was not found to be correlated to the clinical course of the patients. We think that the reason why the number of female patients is similar to the male patients is due to the similar rates of CAD in patients of this age [16]. Owing to the fact that age is the key component of the GRACE score, all the patients had intermediate or high scores. Therefore, high mortality and morbidity rates can be expected. Given that the lowest GRACE score was 123 and the median score was 143 in our study, it is anticipated that the patients would have a mortality rate between 10 and 20% (14, 15). Results revealed that the mortality rate was 16.7% (23) for 6 months and 24.3% (34) for a 2 years period, respectively.

Multivessel disease and chronic total occlusion rates in angiography were remarkable. Almost half of the patients had severe lesions (70%) in more than one epicardial coronary artery. Approximately 20% of the patients had critical (> 95%) lesions in more than one vessel. The number of vessels with ≥ 70% stenosis was not found to have an impact on cardiac and all-cause mortality. There were no complications resulting in death. The prevalence of AF is age-dependent: frequency in the adult population approaches 1–3%, exceeding 15% in people aged 80 years and over [17]. However, we found the frequency of AF to be 12 (8.3%) in our study, which may be due to the limited number of patients.

The most crucial predictor of in-hospital mortality was shock on admission. HFrEF was detected as another risk factor. The level of CRP was a risk factor; however, the OR as 1.02. The low level of OR could be based on the factors like the limited study population, and the low rate of adverse events like intervention site bleeding complications, development of cardiogenic shock or cardiac arrest which patients encountered. Due to these factors, the number of patients with severe CRP elevation has remained low and less CRP elevation was observed. Multiple studies to date have revealed cardiogenic shock on admission and HFrEF to be the most important predictors of in-hospital mortality for younger population (14, 15). Similarly, our findings supported this evidence for very old patients.

Predictors of cardiac death included shock on admission, HFrEF, and CRP levels. Predictors of all-cause mortality were found to be HFrEF, chronic pulmonary disease, CRP levels, and shock on admission in this age group like younger counterparts. The GRACE study indicates age and cardiogenic shock on admission to be the most significant independent predictors of mortality, but there is no discriminative scoring system for patients older than 80 years when calculating the GRACE score, and this patient group has the highest scores. Given the fact that all the patients enrolled in our study were older than 80 years, the GRACE score did not have a marked influence between study group patients. Univariate analysis revealed the correlation between the GRACE score and all-cause mortality; however, the multivariate analysis failed to demonstrate such a connection.

However, mortality rates are in accordance with the GRACE score. Higher mortality rates were reached according to the corresponding GRACE score when the mean mortality rates were assessed.

In sum, for patients with STEMI and NSTEMI older than 80 years, the findings of our study support the routine implementation of CAG and revascularization of suitable lesions with low complication rates- as recommended in current guidelines- if indicated.

## Conclusions

CAG and PCI can be performed with low complication rates in elderly patients. Therefore, aggressive invasive procedures should be performed on these patients because PCI is seems to be a safe treatment option with low complication and mortality rates in this patient group.

## Data Availability

The datasets used and/or analysed during the current study are available from the corresponding author on reasonable request.

## Abbreviations

ACS: Acute coronary syndrome
CAD: Coronary artery disease
CAG: Coronary angiography
CI: Confidence interval
CRP: C-reactive protein
ECG: Electrocardiogram
EF: Ejection fraction
HFrEF: Heart failure with reduced EF
MI: Myocardial infarction
NSTEMI: Non-ST elevated myocardial infarction
OR: Odds ratio
PCI: Percutaneous coronary intervention
STEMI: ST-elevation myocardial infarction
SYNTAX: Synergy between PCI with Taxus and Cardiac Surgery
TLR: Target lesion revascularization
TVR: Target vessel revascularization
